# Efficacy and safety of percutaneous endoscopic decompression via transforaminal and interlaminar approaches for lumbar spine stenosis: Protocol for a systematic review and meta-analysis

**DOI:** 10.1097/MD.0000000000018555

**Published:** 2020-01-03

**Authors:** Yuan Zhen Li, Hong Wei Zhang, Xiao Gang Zhang, Hui Zhang, Li Pan, Xi Yun Zhao, Xue Qian Ning, Zhi Peng Wang

**Affiliations:** a Department of Orthopedic, Affiliated Hospital of Gansu University of Traditional Chinese Medicine; b Clinical College of Chinese Medicine, Guansu University of Traditional Chinese Medicine; c Department of Orthopedic, Gansu Provincial People's Hospital; d Affiliated Hospital of Gansu University of Traditional Chinese Medicine, Lanzhou, China.

**Keywords:** interlaminar, lumbar spinal stenosis, percutaneous endoscopic decompression, systematic review, transforaminal

## Abstract

**Background:**

Lumbar spinal stenosis (LSS) is a common and frequently-occurring disease in the elderly. Percutaneous endoscopic decompression (PED) has become the first choice for the treatment of LSS because of its small wound, mild pain and rapid recovery. The surgical approaches are mainly divided into percutaneous interlaminar approach and transforaminal approach. However, these two surgical approaches have their own advantages, disadvantages and indications. Hence, the present study aims to synthesize the available direct and indirect evidence of transforaminal approach and interlaminar approach to prove their respective advantages and disadvantages.

**Methods:**

The following databases will be searched: Cochrane Library, PubMed, Web of Science, Embase, CNKI, Wanfang data, and China Biomedical Literature Database (CBM). The search dates will be set from the inception to November 2019. Two researchers independently screened the literature, extracted the data and assessed the risk of bias in the included studies. The efficacy outcomes including: Back and Leg Visual Analog Scale (VAS) score, the MacNab criteria, the Oswestry Disability Index (ODI) and Japanese Orthopedic Association (JOA) score. The safety outcomes including: incidence of complications (dura tear, incomplete decompression, reoperation, etc.). The meta-analysis will be conducted using Stata 12.0 software. Grading of Recommendations Assessment, Development and Evaluation (GRADE) will be used to assess evidence quality.

**Results:**

The results of this meta-analysis will be published in a peer-reviewed journal.

**Conclusion:**

The meta-analysis will provide a comprehensive summary of the evidence for 2 approaches to PED in patients with LSS.

**Protocol registration number:**

CRD42019128080.

## 1 Introduction

Lumbar spinal stenosis (LSS) is one of the most common causes of lumbar leg pain, which is common in middle and older people.^[1]^ About one-fifth of elderly patients have symptoms of neurogenic intermittent claudication, and this disease has become the most common cause of spinal surgery in the elderly patients, which significantly affects the activity ability and quality of life of patients.^[2]^ LSS can be divided into central spinal canal stenosis, lateral recess or nerve root canal stenosis and intervertebral foramen stenosis according to the anatomical area.^[3]^ Some patients need surgical treatment, and the traditional open plate removal decompression is good for the effectiveness of the surgery, but the stability of the spine is greatly affected, which is often fixed and integrated. There are disadvantages of surgical trauma, high cost and complications, especially for the elderly.

With the development of the techniques of percutaneous endoscopic lumbar spine surgery, percutaneous endoscopic decompression (PED) has gradually developed into an alternative for the treatment of LSS.^[4–5]^ According to different types, the surgical approaches are mainly divided into percutaneous interlaminar approach and percutaneous transforaminal approach.^[6–9]^ Hypertrophy and hyperplasia of articular process, hypertrophy of yellow ligament and herniation of intervertebral disc are the main reasons for aggravating the clinical symptoms of lumbar spinal stenosis, and adequate decompression is the key to ensure the therapeutic effect.^[10]^

However, their relative efficacy and safety are unclear, and to the best of our knowledge, there is no a study to compare their relative efficacy and safety, then there is a big obstacle for clinicians to select them reasonably. Therefore, we designed a meta-analysis to compare the efficacy and safety of percutaneous endoscopic decompression via transforaminal and interlaminar approaches for LSS, and we hope the results from our present study will provide reference to clinical practice.

## 2 Methods

### 2.1 Study registration

This study protocol has been registered on PROSPERO: CRD42019128080.

### 2.2 Eligibility criteria

#### 2.2.1 Type of study

1) Cohort studies, either randomized controlled trial (RCT) or retrospective study;
2) focusing on the comparison of efficacy or safety between percutaneous endoscopic transforaminal decompression (PETD) and percutaneous endoscopic interlaminar decompression (PEID) in the management of LSS;
3) sufficient data to extract.

#### 2.2.2 Participants

We will include patients with lumbar spinal stenosis were diagnosed using any recognized diagnostic criteria, such as the evidence-based clinical guideline on the diagnosis and treatment of degenerative lumbar spinal stenosis by the North American Spine Society (NASS)^[11]^:

1) lateral recess or nerve root canal stenosis and/or intervertebral foramen stenosis, with or without herniation, have been demonstrated by imaging studies;
2) there were definite radiculopathic symptoms (radialgia, numbness of lower limbs, decreased muscle strength) that were consistent with the imaging data;
3) patients with no clinical efficacy after at least 6 weeks of conservative treatment, including physical therapy and selective nerve root block. But, patients with a lumbar surgery history, infection, tuberculosis, tumors, and other diseases will be excluded.

#### 2.2.3 Interventions

Percutaneous endoscopic decompression (PED), defined as the complete insertion of a thin working sheath through a puncture incision. The working channel endoscope is then placed in the working sheath. The surgical instruments are then introduced through the work channel. The surgical field is always visualized using monitoring systems. The operation was performed under continuous saline irrigation.

Interlaminar PED: The posterior approach was used to reach the intervertebral space with the endoscope with a working channel. The decompression on one side could be completed only by biting off part of the vertebral plates on the cephalic and cendal sides, the medial part of the facet joint and removing the corresponding yellow ligament. The procedure was performed under endoscopic surveillance and continuous 0.9% sodium chloride infusion.

Transforaminal PED: via the lateral approach is suitable for lateral recess stenosis with or without foraminal stenosis. The operation was performed under local anesthesia and conscious sedation. The skin puncture point was usually 10 to 15 cm on the side of the posterior midline, and the skin incision was only 7 mm. The endoscope with working channel was directly entered into the spinal canal through the Kambin safe triangle in the intervertebral foramen, so as to remove the lesion site and effectively relieve pressure.

Percutaneous endoscopic decompression combined with lumbar interbody fusion will be excluded. We will include studies that compared percutaneous endoscopic transforaminal decompression to percutaneous endoscopic interlaminar decompression for LSS.

### 2.3 Outcomes

The efficacy outcomes including: Back and Leg Visual Analog Scale (VAS) score,^[12]^ MacNab criteria,^[13]^ the Oswestry Disability Index (ODI),^[14]^ Japanese Orthopedic Association (JOA) score^[15]^ and operative time, fluoroscopy times, bed time after surgery, hospitalization time. The safety outcomes including: incidence of complications (dura tear, incomplete decompression, reoperation, incidental durotomy, epidural hematoma, headache, infection, recurrence rate). Studies reporting above at least one outcome will be included the present study.

### 2.4 Data source

The following databases will be searched: The Cochrane Library, PubMed, Web of Science, Embase, CNKI, Wanfang data, and China Biomedical Literature Database (CBM). In addition, we will also examine the reference lists of all eligible articles for potential available studies. The search dates will be set from the inception to November 2019. The searching strategy of PubMed is as follows:

#1 “Spinal Stenosis”[Mesh]
#2 “canal stenosis”[Title/Abstract]
#3 (spin∗ adj3 stenosis) [Title/Abstract]
#4 (lumbar adj3 stenosis) [Title/Abstract]
#5 (lateral adj3 stenosis) [Title/Abstract]
#6 (central adj3 stenosis) [Title/Abstract]
#7 (foramin∗ adj3 stenosis) [Title/Abstract]
#8 “neurogenic claudication”[Title/Abstract]
#9 “Radiculopathy”[Mesh]
#10 radiculopathy [Title/Abstract]
#11 “radicular pain”[Title/Abstract]
#12 “lumbar radicular pain”[Title/Abstract]
#13 “Spondylolisthesis”[Mesh]
#14 spondylolisthesis [Title/Abstract]
#15 (lumb∗ adj5 spondyl∗) [Title/Abstract]
#16 #1 OR #2 OR #3 OR #4 OR #5 OR #6 OR #7 OR #8 OR #9 OR #10 OR #11 OR #12 OR #13 OR #14 OR #15
#17 “Endoscopy”[Mesh]
#18 endoscop∗[Title/Abstract]
#19 PED[Title/Abstract]
#20 “Minimally Invasive Surgical Procedures”[Mesh]
#21 #17 OR #18 OR #19 OR #20
#22 (transforaminal OR TF) [Title/Abstract]
#23 (interlaminar OR IL) [Title/Abstract]
#24 #22 OR #23
#25 #21 AND #24
#26 (PELD OR PEID OR PETD OR PIED OR PTED) [Title/Abstract]
#27 #25 OR #26
#28 #16 AND #27

### 2.5 Study selection

**Figure 1:**
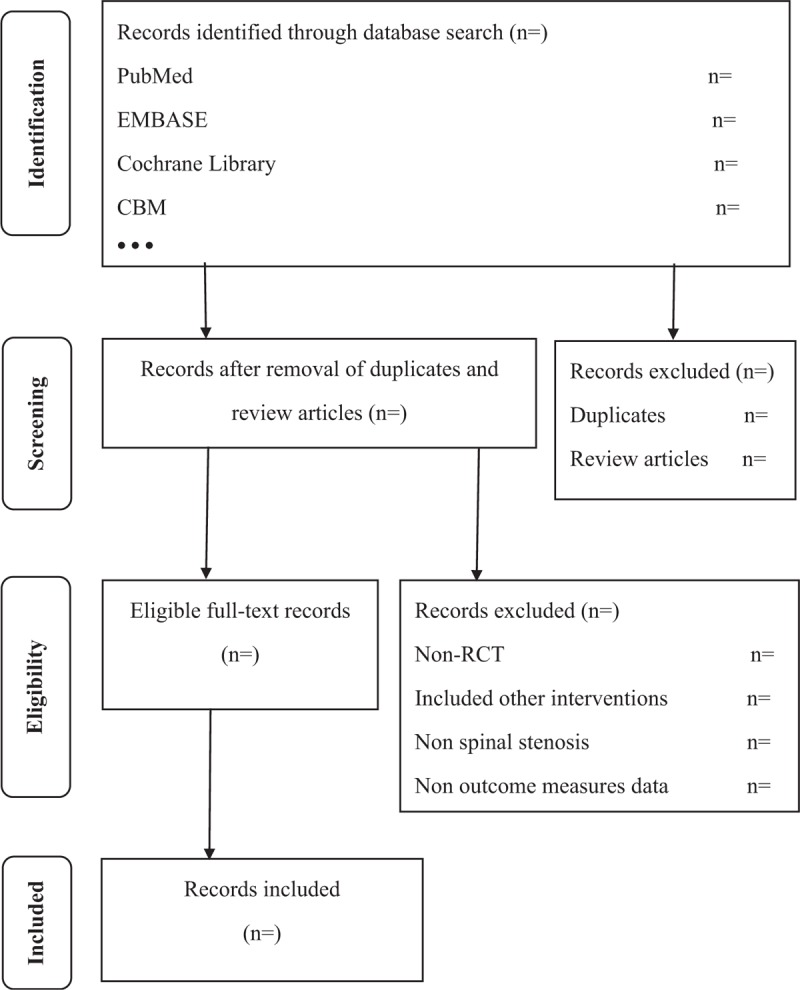
Process of study search and selection.

The research screening process includes 2 stages. First, the titles and abstracts of all articles are reviewed independently by 2 experienced reviewers and appropriate research is conducted according to our eligibility criteria. Second, each full article from the first phase will be downloaded and further reviewed (Fig. 1). Any differences will be resolved through discussion. Detailed data extraction tables will be established with key information including study author, year of publication, study design, sample size, patient characteristics, interventions, treatment options, and results. The third author will examine all extracted information to reduce deviations.

### 2.6 Risk of bias (ROB) assessment

As for ROB, the ROB of retrospective study was assessed according to the Newcastle-Ottawa Scale (NOS),^[16]^ and the risk of bias of RCT was evaluated using the Cochrane Collaboration tool.^[17]^

Two reviewers will independently assess the ROB for all included studies using the Cochrane handbook tool.^[18]^ And this tool consist of 6 domains: random sequence generation, allocation concealment, blind, incomplete outcome data, selective reporting and other bias. The process also will be implemented by 2 reviewers independently and any difference through discussion to reach agreement.

### 2.7 Data extraction and quality assessment

Statistics analysis were calculated with the Stata 12.0 software. Regarding dichotomous, such as sex, complications and MacNab evaluation, the odds ratio (OR) and corresponding 95% confidence interval (CI) were used. As for continuous parameters (e.g., age, operative time, fluoroscopy time, and VAS score), mean difference (MD) or standard mean difference (SMD) were used.

The heterogeneity test was measured by chi-square statistic and I^2^ statistic method. Heterogeneity was qualitatively assessed by comparing the populations baseline, interventions, outcomes, follow-up times and blinding among the included studies. If no serious homogeneity was found (*P* > .10 or I^2^ < 50%), a fixed-effect model was used to analyze the data. If *P* < .10, I^2^ ≥ 50%, indicated a statistical heterogeneity, a random-effect model was used. For continuous outcomes, such as VAS, ODI and operating time, mean difference (MDs) with 95% confidence intervals (CIs) or standardized mean differences with 95%CIs were calculated. For dichotomous outcomes, such as cement leakage rate, risk ratios (RRs) and 95%CIs were calculated. Moreover, in attempting to dissipate any heterogeneity, subgroup analyses were performed based on the characteristics of the treatment duration, gender of participants, and study design. Two-sided *P* values less than .05 were considered statistically significant. Potential publication bias was evaluated using funnel plot.

### 2.8 Quality of evidence

The quality of evidence for all outcomes will be assessed using the Grading of Recommendations Assessment, Development and Evaluation (GRADE)^[19]^ mainly considerations including: risk of bias, inaccuracy, inconsistency, indirectness, publication bias, and results of assessment will be graded four levels: very low, low, moderate, and high level.

## 3 Discussion

To date, this is the first meta-analysis to compare the efficacy and safety of percutaneous endoscopic decompression via transforaminal and interlaminar approaches for LSS. Therefore, the current meta-analysis will fill this gap according to the Cochrane handbook and the PRISMA extension statement. The study will provide an available direct and indirect evidence on the transforaminal and interlaminar approaches for LSS, and to generate a treatment ranking based on their efficacy and safety outcomes. This protocol is designed according to the PRISMA-P,^[20]^ which is used for reporting systematic review protocol, whether or not a meta-analysis is performed.

## Author contributions

**Data curation**: Yuan Zhen Li, Hong Wei Zhang, Zhi Peng Wang.

**Methodology**: Xiao Gang Zhang, Hui Zhang, Xi Yun Zhao.

**Resources**: Yuan Zhen Li, Zhi Peng Wang.

**Software**: Zhi Peng Wang, Li Pan, Xue Qian Ning.

**Writing – original draft**: Yuan Zhen Li, Zhi Peng Wang.

**Writing – review & editing**: Yuan Zhen Li, Hong Wei Zhang, Zhi Peng Wang.

## References

[1] Ahn Y Percutaneous endoscopic decompression for lumbar spinal stenosis. Expert Rev Med Devices 2014;11:605–16. 25033889 10.1586/17434440.2014.940314

[2] Choi WS Oh CH Ji GY Spinal canal morphology and clinical outcomes of microsurgical bilateral decompression via a unilateral approach for lumbar spinal canal stenosis[J]. Eur Spine J 2014;23:991–8. 24292345 10.1007/s00586-013-3116-7

[3] Ahn Y Keum HJ Lee SG Transforaminal endoscopic decompression for lumbar lateral recess stenosis: an advanced surgical technique and clinical outcomes. World Neurosurg 2019;pii: S1878-8750(19)30320-1. 10.1016/j.wneu.2019.01.209 30763754

[4] Ruetten S Komp M Hahn P Decompression of lumbar lateral spinal stenosis: full-endoscopic, interlaminar technique. Oper Orthop Traumatol 2013;25:31–46. 23371002 10.1007/s00064-012-0195-2

[5] Yang J Wu H Kong Q Full endoscopic transforaminal decompression surgery for symptomatic lumbar spinal stenosis in geriatric patients. World Neurosurg 2019;127:e449–59. 30922895 10.1016/j.wneu.2019.03.171

[6] Wen B Zhang X Zhang L Percutaneous endoscopic transforaminal lumbar spinal canal decompression for lumbar spinal stenosis. Medicine (Baltimore) 2016;95:e5186. 27977571 10.1097/MD.0000000000005186 PMC5268017

[7] Torudom Y Dilokhuttakarn T Two portal percutaneous endoscopic decompression for lumbar spinal stenosis: preliminary study. Asian Spine J 2016;10:335–42. 27114776 10.4184/asj.2016.10.2.335 PMC4843072

[8] Li ZZ Hou SX Shang WL Percutaneous lumbar foraminoplasty and percutaneous endoscopic lumbar decompression for lateral recess stenosis through transforaminal approach: Technique notes and 2 years follow-up. Clin Neurol Neurosurg 2016;143:90–4. 26907998 10.1016/j.clineuro.2016.02.008

[9] Kapetanakis S Gkantsinikoudis N Thomaidis T The role of percutaneous transforaminal endoscopic surgery in lateral recess stenosis in elderly patients. Asian Spine J 2019 638–47. 30909678 10.31616/asj.2018.0179 PMC6680028

[10] Li Y Wang B Wang S Full-endoscopic decompression for lumbar lateral recess stenosis via an interlaminar approach versus a transforaminal approach 2019;128:e632–8. 10.1016/j.wneu.2019.04.221 31054348

[11] Kreiner DS Shaffer WO Baisden JL An evidence-based clinical guideline for the diagnosis and treatment of degenerative lumbar spinal stenosis (update). Spine J 2013;13:734–43. 23830297 10.1016/j.spinee.2012.11.059

[12] Zanoli G Strömqvist B Jönsson B Visual analog scales for interpretation of back and leg pain intensity in patients operated for degenerative lumbar spine disorders. Spine (Phila Pa 1976) 2001;26:2375–80.11679824

[13] Macnab I Negative disc exploration. An analysis of the causes of nerve-root involvement in sixty-eight patients. J Bone Joint Surg Am 1971;53:891–903. 4326746

[14] Fairbank JC Pynsent PB The Oswestry Disability Index. Spine (Phila Pa 1976) 2000;25:2940–52. 11074683 10.1097/00007632-200011150-00017

[15] Shirado O Doi T Akai M An outcome measure for Japanese people with chronic low back pain: an introduction and validation study of Japan Low Back Pain Evaluation Questionnaire. Spine (Phila Pa 1976) 2007;32:3052–9. 18091501 10.1097/BRS.0b013e31815cda68

[16] Stang A Critical evaluation of the NewcastleOttawa scale for the assessment of the quality of nonrandomized studies in meta-analyses. Eur J Epidemiol 2010;25:603–5. 20652370 10.1007/s10654-010-9491-z

[17] Higgins JPT Green S Cochrane Handbook for Systematic Reviews of Interventions, Version 5. 1. 0. London: The Cochrane Collaboration; 2011.

[18] Higgins JPT Altman DG Sterne JAC Chapter 8: Assessing risk of bias in included studies. In: Higgins, J.P.T., Green, S., editors. Cochrane Handbook for Systematic Reviews of Interventions version 5.1.0 (updated March 2011): The Cochrane collaboration. Available from www.chochrane-handbook.org 2011:243–96.

[19] Shamseer L Moher D Clarke M Preferred reporting items for systematic review and meta-analysis protocols (PRISMA-P) 2015: elaboration and explanation. BMJ 2015;349:g7647. 10.1136/bmj.g7647 25555855

[20] Hutton B Salanti G Caldwell DM The PRISMA extension statement for reporting of systematic reviews incorporating network meta-analyses of health care interventions: checklist and explanations. Ann Intern Med 2015;162:777–84. 26030634 10.7326/M14-2385

